# Weibull Analysis of Fracture Test Data on Bovine Cortical Bone: Influence of Orientation

**DOI:** 10.1155/2013/639841

**Published:** 2013-12-09

**Authors:** Morshed Khandaker, Stephen Ekwaro-Osire

**Affiliations:** ^1^z Department of Engineering and Physics, University of Central Oklahoma, Edmond, OK 73034, USA; ^2^Department of Mechanical Engineering, Texas Tech University, Lubbock, TX 79409, USA

## Abstract

The fracture toughness, *K*_IC_, of a cortical bone has been experimentally determined by several researchers. The variation of *K*_IC_ values occurs from the variation of specimen orientation, shape, and size during the experiment. The fracture toughness of a cortical bone is governed by the severest flaw and, hence, may be analyzed using Weibull statistics. To the best of the authors' knowledge, however, no studies of this aspect have been published. The motivation of the study is the evaluation of Weibull parameters at the circumferential-longitudinal (CL) and longitudinal-circumferential (LC) directions. We hypothesized that Weibull parameters vary depending on the bone microstructure. In the present work, a two-parameter Weibull statistical model was applied to calculate the plane-strain fracture toughness of bovine femoral cortical bone obtained using specimens extracted from CL and LC directions of the bone. It was found that the Weibull modulus of fracture toughness was larger for CL specimens compared to LC specimens, but the opposite trend was seen for the characteristic fracture toughness. The reason for these trends is the microstructural and extrinsic toughening mechanism differences between CL and LC directions bone. The Weibull parameters found in this study can be applied to develop a damage-mechanics model for bone.

## 1. Introduction

**Figure 1:**
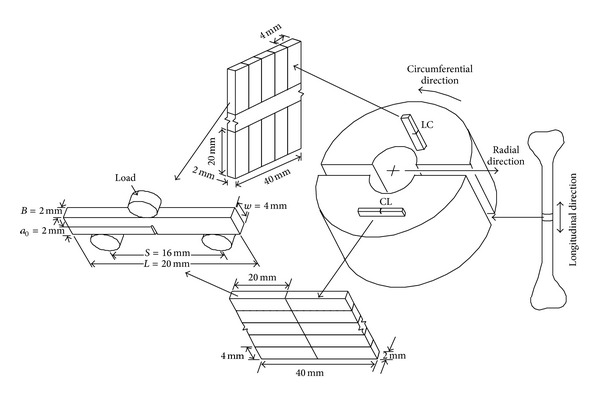
Preparation of 3-pt bend specimen in two directions: circumferential-longitudinal (CL) and longitudinal-circumferential (LC), where the first letter of the specimen name refers to the normal direction of the created crack plane and the second letter refers to the expected direction of crack propagation.

Bone is anisotropic material. The fracture toughness of bone varies depending on sampling site and the initial crack orientation of fracture test samples with respect to the applied load. Researchers prepared specimen in different orientations to measure fracture toughness [1–4]. The orientation of the specimen used in the fracture test was based on fracture propagation direction with respect to the long axis of the bone. According to crack orientation, there were two types of specimen considered for the measurement of fracture toughness: longitudinal cracking specimen and transverse cracking specimen. In the longitudinal cracking specimen, a crack propagates parallel to the long axis that is, along the collagen fiber through the bone matrix, whereas in the transverse cracking specimen, a crack propagates normal to the long axis, that is, across the long axis. In this study, circumferential-longitudinal (CL) and longitudinal-circumferential (LC) direction specimen are categorized as longitudinal cracking and transverse cracking specimen, respectively (Figure 1). Crack orientation of a specimen was classified according to crack plane during fracture toughness testing. The first letter represents a normal direction to the created crack plane; the second letter represents the expected direction of crack propagation [5].

There are many reports of plane strain mode I fracture toughness, *K*_IC_, in different bone materials (cortical and trabecular bones) obtained under both quasistatic and dynamic loading conditions [6–9]. Typically, there is a large scatter in *K*_IC_ values because of nonuniformity in specimen microstructure, size, shape, and initial crack length. Since the standard deviation, in the measurement of fracture toughness, has a similar magnitude to the average value, a single value fracture toughness is not a reliable parameter to predict fracture toughness for bone materials [10]. Fracture of a bone occurs where it is structurally weakest (at the Haversian and Volkmann [10] canals). Hence, it is appropriate to use Weibull statistics [11, 12] to analyze fracture test data. Assuming that the fracture toughness distribution is describable using the two-parameter Weibull equation, the probability distribution function is given by [13]

(1) $$\begin{array}{c} F\left(K_{\text{IC}}\right)=1-e^{-{\left(K_{\text{IC}}/K_{0}\right)}^{m}}, \end{array}$$

where *K*_IC_ is the fracture toughness and *F*(*K*_IC_) is the failure probability. In (1), *m* is the Weibull modulus, which indicates scatterness of test data, and *K*_0_ is the characteristic fracture toughness or scale parameter (fracture toughness value that includes 63.2% of the test data, when *K*_IC_ = *K*_0_). The objective of the present work was to determine the influence of the orientation of a cortical bone from which the test specimen was extracted on the values of the Weibull modulus and the scale parameter of *K*_IC_.

## 2. Materials and Methods

### 2.1. Specimen Configuration

Power analysis was conducted to determine the appropriate test samples using the mean and standard deviation of fracture toughness of cortical bone in longitudinal and transverse direction found from Lucksanasombool et al. [1]. The analysis found that over 24 samples provide a 95% confident estimate of fracture toughness for CL and LC specimens with an error of 0.04 from their population mean. Therefore, more than 24 samples of CL and LC specimens were made during this study. Fifty-three 20 mm × 4 mm × 2 mm dimension single edge-notch bend (SENB) specimens were prepared from cortical bone harvested from the femur of an animal of 18 months old or less, with 28 and 25 cuts from the CL and LC orientations, respectively (Figure 1). The initial notch of the specimens was 2 mm. The dimensions of the specimens conformed to ASTM E 399 standard [14].  Table 1 shows the dimensions of CL and LC specimens with their mean and standard deviation. The initial crack length was created at the center of the specimens by a Buehler Isomet 11-1180-160 diamond saw cutter with a thickness of 0.125 mm. Nikon's SMZ-2500 stereo microscope was used to view the topography of the specimens and measure the dimensions of the specimens.

### 2.2. Experiment

**Figure 2:**
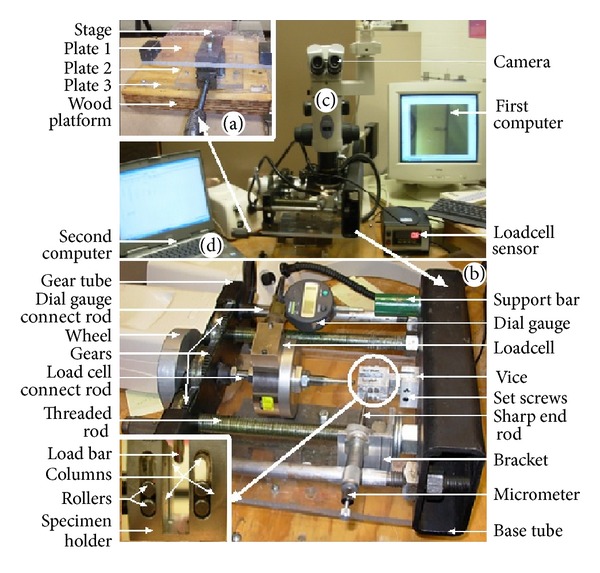
The fabricated experimental setup used in the study. The setup consists of (a) stage mechanism, (b) specimen loading, holding, aligning, and measuring systems, (c) camera system, and (d) computers.

A custom made three-point bending test apparatus (see Figure 2) was used in this study. The bone specimens were supported horizontally by two rollers located 16 mm apart, which is the spun length, *S*, for the SENB test. A load cell (FUTEK, model L2920) with a digital display (FUTEK, model IBT 500) was used to measure the indention load that was applied at the midspan of the bone. A digital displacement gage (Mitutoyo, Series 543) was used to measure the load point displacement. The data obtained was used to generate force versus displacement graphs. The crack initiation and propagation were observed using a stereomicroscope (Nikon's SMZ-2500), focused at the midpoint of the bone during the tests.

### 2.3. Data Analysis

The critical loads for rapture, *P*_cr_, for the CL and the LC samples were extracted from the load-displacement graph. The value of *K*_IC_ was calculated using the following expression [15]:

(2) $$\begin{array}{c} K_{\text{IC}}=\frac{P_{\text{cr}}Y}{BW^{0.5}}, \end{array}$$

where *B* is the specimen thickness, *W* is the width, and *Y* is the shape function at the initial crack length, *a*_0_. For three-point bend testing, the values of *Y*were calculated using [16]

(3) Y=(α1−α)2(5.58−19.57α+36.82α2       −34.95α3+12.77α4),

where *α* is the normalized crack length defined as *α* = *a*_0_/*W*. The Weibull method was used to calculate the Weibull parameters (*m* and *K*_0_) of the fracture toughness, *K*_IC_, data for CL and LC specimens. According to the method, (1) can be expressed as

(4) $$\begin{array}{c} \ln\left[\ln\left(\frac{1}{1-F\left(K_{\text{IC}}\right)}\right)\right]=m\ln K_{\text{IC}}-m\ln K_{0}. \end{array}$$

After sorting and numbering the *K*_IC_ values of all the CL and LC specimens in ascending order, the failure probabilities of *F*(*K*_IC_) were estimated by (*j* − 0.3)/(*N* + 0.4), where *j* is the rank number of the specimen's fracture toughness and *N* is the specimen sample size (*N* = 28 for CL specimen and *N* = 25 for LC specimen). The *F*(*K*_IC_) and *K*_IC_ data were used to generate the Weibull statistic graphs [lnln(1/(1 − *F*(*K*_IC_))) versus ln⁡(*K*_IC_)]. Univariate regression analyses of the *F*(*K*_IC_) against the *K*_IC_ data was conducted to calculate *m* and *K*_*o*_ using Microsoft Excel Weibull analysis add-on [17]. The Weibull modulus was calculated directly from the slope of the Weibull statistic graphs. A one-way ANOVA analysis was used to compare Weibull modulus values as a function of orientation for bone specimens. The estimate for the scale parameter was calculated using

(5) $$\begin{array}{c} K_{0}=e^{\left|\left(-b/m\right)\right|}, \end{array}$$

where *b* was calculated as −*m*ln⁡*K*_0_, which is the intercept of the Weibull graph. The Weibull statistical model equation (1) was applied to the fracture toughness (*K*_0_ = *K*_IC_) to obtain the value of probability of failure of fracture toughness.

## 3. Results and Discussion

**Figure 3:**
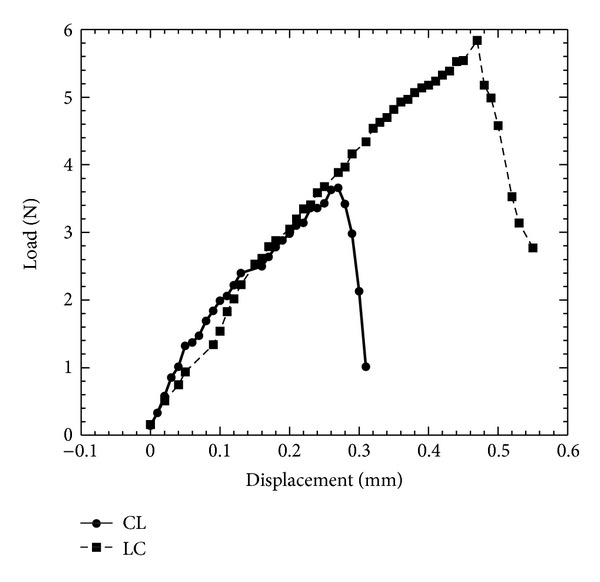
Typical load-displacement curves of a CL and LC specimen.

**Figure 4:**
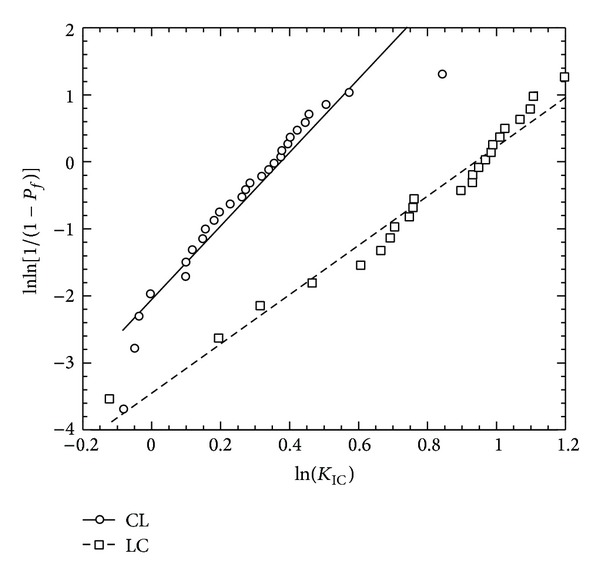
Weibull plots of fracture toughness.

**Figure 5:**
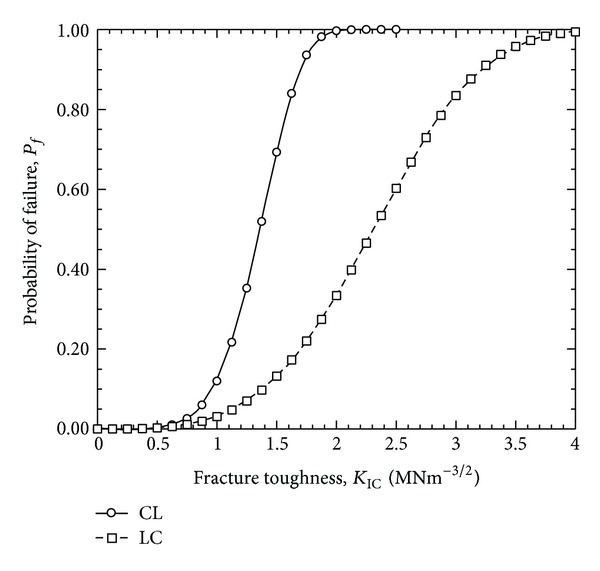
Cumulative distribution function of fracture toughness.

The results are presented in Figures 3 to 5 and Tables 2 to 3. The comparison of the load-displacement plots of a CL and LC specimen is shown in Figure 3. This figure shows that the slope of the load versus the displacement value is steeper at the initial stage of the experiment than at failure condition. This result means a higher load is required at the initial stage than later near failure for the same load point displacement. The increment of the load with displacement behaved linearly until the onset of cracking which is observed in the figure by the nonlinearity (fracture initiation) point of the load-displacement curve. Also, Figure 3 shows that for the same notch depth ratio, the LC specimen requires a higher initial fracture load than CL load.  Figure 4 shows the Weibull statistic graphs [lnln(1/(1 − *F*(*K*_IC_))) versus ln⁡(*K*_IC_)]. The regression model coefficients for the least square fit of Weibull graphs for both specimens are given in Table 2. Results show that specimen direction had significant regressions of the transformed failure probability (*F*(*K*_IC_)) ((4)) against ln⁡(*K*_IC_) (*P* < 0.001, Figure 4), demonstrating that the two-parameter Weibull model is applicable for the analysis.

Figure 5 shows the cumulative distribution functions of the fracture toughness value for the CL and LC specimens. Figure 5 also shows a wider range of fracture stress for the LC specimens compared to the CL specimens. Individual Weibull strength moduli, *m*, and characteristic fracture toughness, *K*_0_, were calculated for each group (Table 3). The Weibull strength moduli were significantly different (ANOVA, *P* < 0.001) between the specimen groups, as expected because the slopes are related to the specimens' material failure properties. The Weibull modulus of the CL specimens is higher than that of the LC specimen. This means that the scatterness of the fracture toughness value was lower for the CL specimen compared to the LC specimen. Since the scale parameter of the LC specimens is higher than that of the CL specimens, one expects to find that the fracture toughness is higher for the LC specimens than that for the CL specimens for a particular failure probability (Figure 5).

Different distribution of structural types in front of the propagation crack in CL and LC directions specimens can be the reason for the difference of Weibull parameters of *K*_IC_ between the two groups. This explanation is in agreement with Lipson and Katz [18]. The authors reported a different distribution of the structural types within different locations of the same bovine femur. They found that a different level of osteonal remodeling is directly related to the pattern of mechanical stress at the various fracture sites. The crack propagation in the CL specimens was parallel to the collagen fibers through the bone matrix, whereas in the LC specimen, the crack propagated across the collagen fibers. For LC specimens, the variation of the mechanical stress at the tip of the crack is due to high variation of the bone matrix and/or mineralized collagen fibrils and/or osteonal systems in the transverse direction. On the other hand, for CL specimens, the variation of the mechanical stress at the tip of the crack is due to variation of structural differences of the cement line, the boundary between secondary osteons and the surrounding lamellar matrix. The difference between the extrinsic toughening behavior for the LC and the CL specimens may be the source of a larger characteristic fracture toughness value in the LC specimens compared to the CL specimens. According to Ritchie et al. [5], extrinsic toughening in front of the propagating crack tip in LC specimen occurs due to the collagen fiber bridging, crack deflection due to the cement line, uncracked ligament bridging, and microcracking, whereas extrinsic toughening in front of the crack tip in CL specimen occurs mainly by microcracking and uncracked ligament bridging.

There are no publications on Weibull parameters of fracture toughness data for bovine cortical bone in CL and LC to compare the results of this investigation. However, Pithioux et al. [8] applied the Weibull distribution to bovine compact bone tension failure under quasistatic loading. Dog bone shape tensile specimen samples were used for the study. The direction of fracture of the specimen was the same as the CL specimen used in this study. The Weibull tensile strength modulus (scatterness of tensile strength) was found to be 5.77 by Pithioux et al. [8], which is in close agreement with the Weibull modulus of fracture toughness in this study (5.48). This difference was reasonable since the bone type, age, size, loading, shape, and measurement properties used in this study were different from those of the test specimen used by Pithioux et al. [8].

The results of this study can be utilized to quantify the role of bone toughening mechanism on the failure probability of extremely diverse range of biomedical and nonbiomedical applications and has potential use for orthopedic bone cement. Also, a failure model that considers the physiological process like remodeling, adaptation can be investigated. This study will enable biomedical researchers to predict more effectively the microdamage associated with bone damage and design bone-implant systems.

## 4. Conclusion

The aim of this paper was to compare the bovine cortical bone fracture toughness in two different directions: CL and LC. Weibull statistical law was used to analyze the fracture failure variation and characteristic fracture toughness values of CL and LC specimens. The results of this study are as follows.

1. The individual fracture toughness data for each direction of specimen tested follow the two-parameter Weibull distribution. Each distribution was characterized by a different Weibull modulus.
2. LC direction cortical bone possesses greater fracture toughness than LC direction specimen for a particular failure probability level.
3. There is a statistically significant difference of Weibull modulus and characteristic fracture toughness for specimens of different orientation. The observed variability in the fracture of bovine cortical bone in CL and LC directions was explained by microstructural variables depending on the direction.

## Figures and Tables

**Table 1:** Dimension of the prepared specimen. In the table, *S* is the spun length.

Specimen type	Sample number, *N*	Length, *L* (mm)	Width, *W* (mm)	Thickness, *B* (mm)	Initial crack length *a*_0_ (mm)	*S*/*W*	W/B	a_0_/W
CL	28	19.06 ± 0.26	3.70 ± 0.29	2.26 ± 0.11	1.94 ± 0.18	4.34 ± 0.37	1.65 ± 0.20	0.52 ± 0.03
LC	25	19.88 ± 0.34	3.73 ± 0.14	2.09 ± 0.22	1.89 ± 0.12	4.29 ± 0.17	1.81 ± 0.20	0.51 ± 0.03

**Table 2:** Multiple regression model parameters for predicting fracture toughness (*P* < 0.001).

Regression statistics parameters	CL	LC
Multiple *R*	0.949	0.984
*R* ^2^	0.901	0.969
Adjusted *R* ^2^	0.897	0.967
Standard error	0.383	0.214

**Table 3:** Statistical parameters of fracture toughness test.

Specimen type	Number of specimen	Weibull parameters	
		Fracture toughness, *K*_IC_	
		Modulus, *m*	Scale parameter, *K*_0_ (MN·m^−3/2^)
CL	28	5.48	1.45
LC	24	3.67	2.56
